# Segmenting vegetation from UAV images via spectral reconstruction in complex field environments

**DOI:** 10.1016/j.plaphe.2025.100021

**Published:** 2025-03-01

**Authors:** Zhixun Pei, Xingcai Wu, Xue Wu, Yuanyuan Xiao, Peijia Yu, Zhenran Gao, Qi Wang, Wei Guo

**Affiliations:** a State Key Laboratory of Public Big Data, College of Computer Science and Technology, Guizhou University, Guiyang, 550025, China; b State Key Laboratory of Green Pesticide, Guizhou University, Guiyang 550025, China; c Graduate School of Agricultural and Life Sciences, The University of Tokyo, Tokyo, 188-0002, Japan; d New Rural Development Research Institute, Guizhou University, Guiyang, 550025, China

**Keywords:** Segmentation, Spectral reconstruction, UAV field images

## Abstract

Segmentation of vegetation remote sensing images can minimize the interference of background, thus achieving efficient monitoring and analysis for vegetation information. The segmentation of vegetation poses a significant challenge due to the inherently complex environmental conditions. Currently, there is a growing trend of using spectral sensing combined with deep learning for field vegetation segmentation to cope with complex environments. However, two major constraints remain: the high cost of equipment required for field spectral data collection; the availability of field datasets is limited and data annotation is time-consuming and labor-intensive. To address these challenges, we propose a weakly supervised approach for field vegetation segmentation by using spectral reconstruction (SR) techniques as the foundation and drawing on the theory of vegetation index (VI). Specifically, to reduce the cost of data acquisition, we propose SRCNet and SRANet based on convolution and attention structure to reconstruct multispectral images of fields, respectively. Then, borrowing from the VI principle, we aggregate the reconstructed data to establish the connection of spectral bands, obtaining more salient vegetation information. Finally, we employ the adaptation strategy to segment the fused feature map using a weakly supervised method, which does not require manual labeling to obtain a field vegetation segmentation result. Our segmentation method can achieve a Mean Intersection over Union (MIoU) of 0.853 on real field datasets, which outperforms the existing methods. In addition, we have open-sourced a dataset of unmanned aerial vehicle (UAV) RGB-multispectral images, comprising 2358 pairs of samples, to improve the richness of remote sensing agricultural data. The code and data are available at ​https://github.com/GZU-SAMLab/VegSegment_SR, and ​http://sr-seg.samlab.cn/.

## 1 Introduction

With rapid population growth and consequent environmental degradation, improving crop yields and optimizing growing conditions have become an increasingly pressing issue [1]. According to the latest projections by the United Nations, the global population will grow to around 8.5 billion by 2030, increasing the demand for food [2]. The increase in food production cannot be achieved without monitoring the growing process of crops. Environmental fluctuations in agricultural fields are key determinants of plant growth, especially crops, and thus real-time field monitoring is important in crop cultivation. Traditional field monitoring methods are manual-driven, generally resource-intensive and expensive [3]. In addition, the constraints of complex field environments (e.g., real fields of different time periods, different crops, and different cropping practices) make manual measurements difficult to balance. Nowadays, the convergence of different disciplines facilitates the emergence of smart agriculture via computer vision and remote sensing technologies, which offer many efficient, economical, and harmless solutions for crop monitoring [[4], [5], [6], [7], [8]]. Specifically, computer vision and remote sensing measurements can be used to process collected image data of field vegetation to efficiently monitor and analyze crop growth in the field [9,10].

In field vegetation monitoring, segmentation methods incorporating spectral data have become mainstream [[11], [12], [13]]. Compared to RGB images, spectral images are able to capture more spectral information beyond the visible spectral range, especially in the near-infrared (NIR) band, and are more sensitive to the reflective characteristics of vegetation, which gives them a significant advantage in distinguishing vegetation from other background elements. As a result, these segmentation methods can accurately and quickly detect fallen, weed, and wilted plants by focusing precisely on the plants themselves [14]. For instance, Polewski et al. [15] use convolutional networks to segment fallen trees in aerial color infrared images. Sahin et al. [16] utilize U-Net for weed and crop segmentation by fusing multiple spectral data. Serouart et al. [17] propose the semantic segmentation of field data using U-Net and SVM to distinguish between fresh vegetation and senescent vegetation. Zhang et al. [18] report on the analysis of cabbage breeding using models to process multispectral unmanned aerial vehicle (UAV) images and measure the phenotypic data of individual cabbages. Liu et al. [19] design a multimodal hierarchical fusion method based on the attention mechanism for the instance segmentation of tomato main stems. A number of studies have demonstrated the ability of segmentation methods based on spectral data to cope with complex field monitoring. However, spectral data acquisition is expensive and traditional vegetation segmentation methods need a complex data labeling process.

**Fig. 1:**
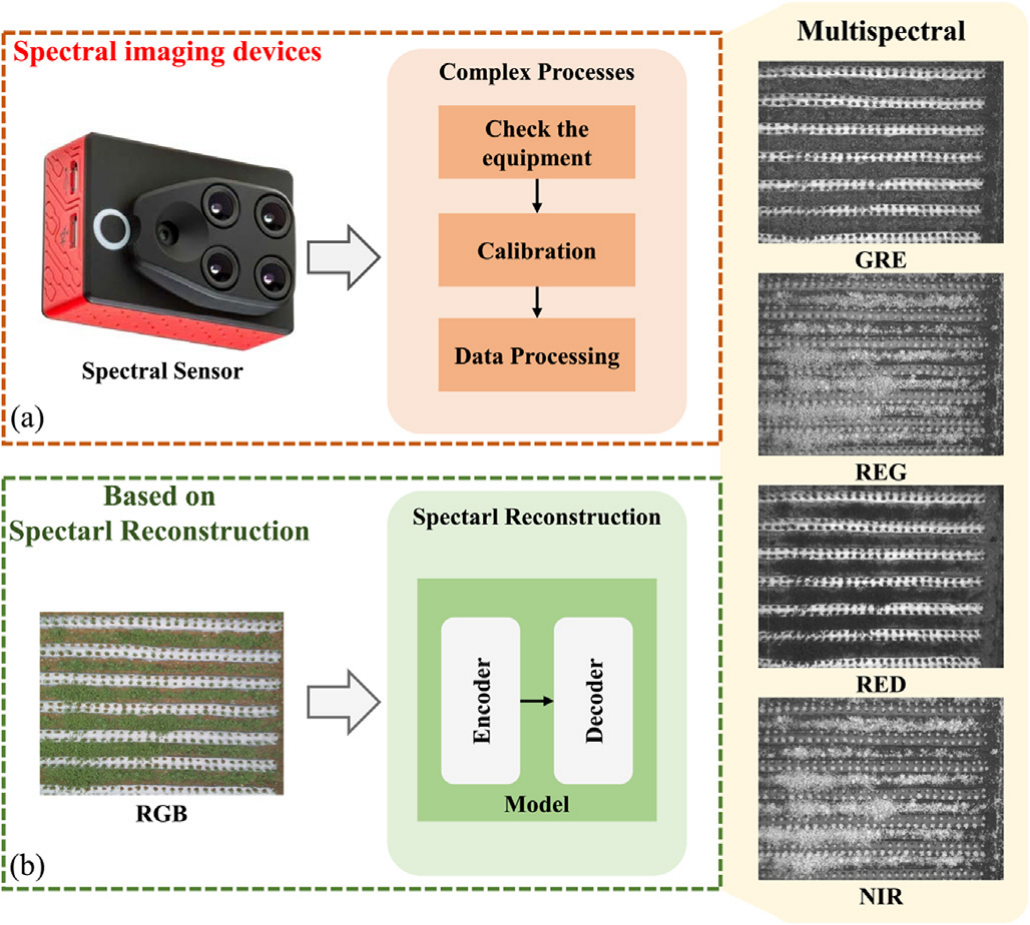
Comparison of spectral imaging methods. (a) Traditional multispectral data acquisition via spectral imaging devices. (b) Spectral data acquisition based on SR methods via RGB image.

In search of more economical ways, researchers explore spectral reconstruction (SR) techniques to acquire low-cost, high-quality spectral data from RGB [20,21]. As shown in Fig. 1, the SR method does not require a complex imaging process only requires a trained model to be able to obtain spectral data. In recent popular studies, spectral reconstruction is one of the tasks of the New Trends in Image Restoration and Enhancement (NTIRE) [[22], [23], [24]] competition. Xiong et al. [25] propose the HSCNN deep learning framework for hyperspectral reconstruction from RGB and compressed measurements. Shi et al. [26] propose a convolutional neural network(CNN)-based model for SR. Cai et al. [27] introduce the attention mechanism [28] into the SR task and achieved good results. The above methods greatly facilitate the acquisition of spectral data, and they are essential for agricultural field monitoring. In addition, some researchers have applied SR techniques to crop monitoring. For example, Zhao et al. [29] utilize SR techniques to predict the quality characteristics of tomatoes from the reconstructed data. Further, Zhao et al. [30] demonstrate the enhancement of SR techniques for phenotyping plants in unmanned aerial vehicle. However, although SR technology has shown some potential for crop monitoring, it has not yet been able to cope with the complexity and diversity of real-field environments, due to the lack of interconnection between the field spectral information and the vegetation phenotype.

Building upon these ideas, we propose a novel weakly supervised approach for UAV field vegetation analysis that leverages spectral reconstruction for plant phenotype segmentation. Compared with traditional methods, our method can significantly reduce the cost of spectral data acquisition while not requiring segmentation labeling. High-quality and high-precision vegetation phenotype segmentation data are obtained while reducing the cost. In this article, we first propose SRCNet and SRANet for realizing multispectral reconstruction from RGB images and then apply the reconstruction results for vegetation segmentation. More specifically, the SR model contains an encoder and decoder for image feature extraction and spectral recovery, respectively. Then, the proposed model learns the implied relationship between RGB and spectral data in the first stage and reconstructs RGB images into multispectral images. In addition, the reconstructed data are processed in the second stage based on the reconstructed spectral attributes combined with vegetation index (VI), and thus segmenting the field vegetation phenotypic data. Finally, the obtained segmented vegetation phenotype data can optimize the vegetation monitoring process. Extensive experiments have demonstrated that our proposed method can cope with a variety of complex environments and achieve excellent results.

The specific contributions are as follows:

• We propose an innovative RGB-based segmentation method to reconstruct connections between spectral information and vegetation phenotype. This is a weakly supervised method that does not require data annotation.
• Our method can be applied to real field data with low overhead, few iterations, and superior performance to overcome complex external environmental factors.
• We have open-sourced an RGB-Multispectral dataset to facilitate smart agriculture development. Code and data are available at https://github.com/GZU-SAMLab/VegSegment_SR, and http://sr-seg.samlab.cn/.

## 2 Materials and methods

### 2.1 In-field data acquisition

**Fig. 2:**
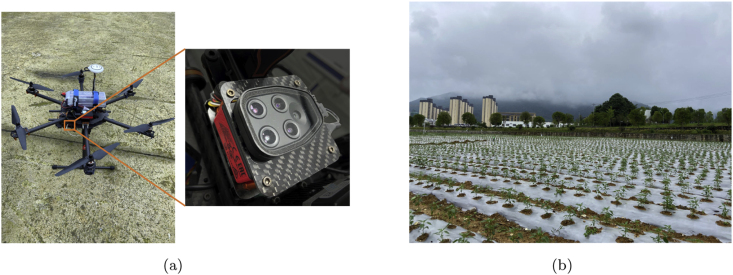
The data acquisition environment and the devices. (a) Data acquisition devices. (b) Field environment.

The majority of equipment related to multispectral agricultural data acquisition uses multi-lens type multispectral cameras. In our study, we use Parrot Sequoia visible-multispectral sensors aboard UAV to collect field data, as shown in Fig. 2(a). The Parrot Sequoia visible-multispectral is often applied in precision agriculture, which has a 16 Mpx RGB sensor for capturing visible light images and four 1.2 Mpx monochrome sensors for green (550 ​nm wavelength, 40 ​nm bandwidth), red (660 ​nm wavelength, 40 ​nm bandwidth), red-edge (735 ​nm wavelength, 40 ​nm bandwidth) and near-infrared (790 ​nm wavelength, 40 ​nm bandwidth). To extend the image acquisition range, the Parrot Sequoia visible-multispectral sensor uses a lens with fisheye distortion to enhance the image view, which makes subsequent image processing challenging due to this distortion. We fix the sensor on a six-rotor UAV and fly it around the field to collect data.

To increase the variety of data, there is no fixed uniform altitude at which the UAV flies, and we collect at multiple locations and time periods. Due to the ups and downs of the field ground, the relative altitude of each group of flights is about 10 ​m. By collecting data at different altitudes, we can simulate a more complex and diverse actual field environment, ensuring that the model is still adaptable and stable in response to different viewing angles and resolutions. The complete field data is collected three times and contains a total of four planted crops, and the field environment is shown in Fig. 2(b). The first collection is conducted in two time periods, the first one on the afternoon of June 11, 2022, with cloudy weather, and the second one on the morning of June 12, 2022, with cloudy weather. The main crop collected this time is pepper, in which pepper plants evenly and sparsely, with weeds and insulation films distributed in the field, etc. The second collection is conducted on the morning of July 7, 2022, in sunny weather. The main crop collected this time is sorghum, in which sorghum plants densely. The third collection is conducted at noon on July 21, 2022, in sunny weather. The main crops collected this time are sorghum, rice, and tomato, which the crops distributed densely. Due to the interference of external factors when collecting data, some data cannot be used such as data during UAV takeoff and landing, data without valid information, etc. Therefore, it is necessary to filter the collected data and perform operations such as correction and alignment.

### 2.2 Data preprocessing

Since the Parrot Sequoia spectral sensor is a multi-lens device that uses a fisheye lens to increase the image capture area, the acquired image data has distortions and pixel point offset among bands, so the captured field data cannot be used directly. Therefore, we need to perform data pre-processing for the subsequent model training, including three steps.

**Fig. 3:**
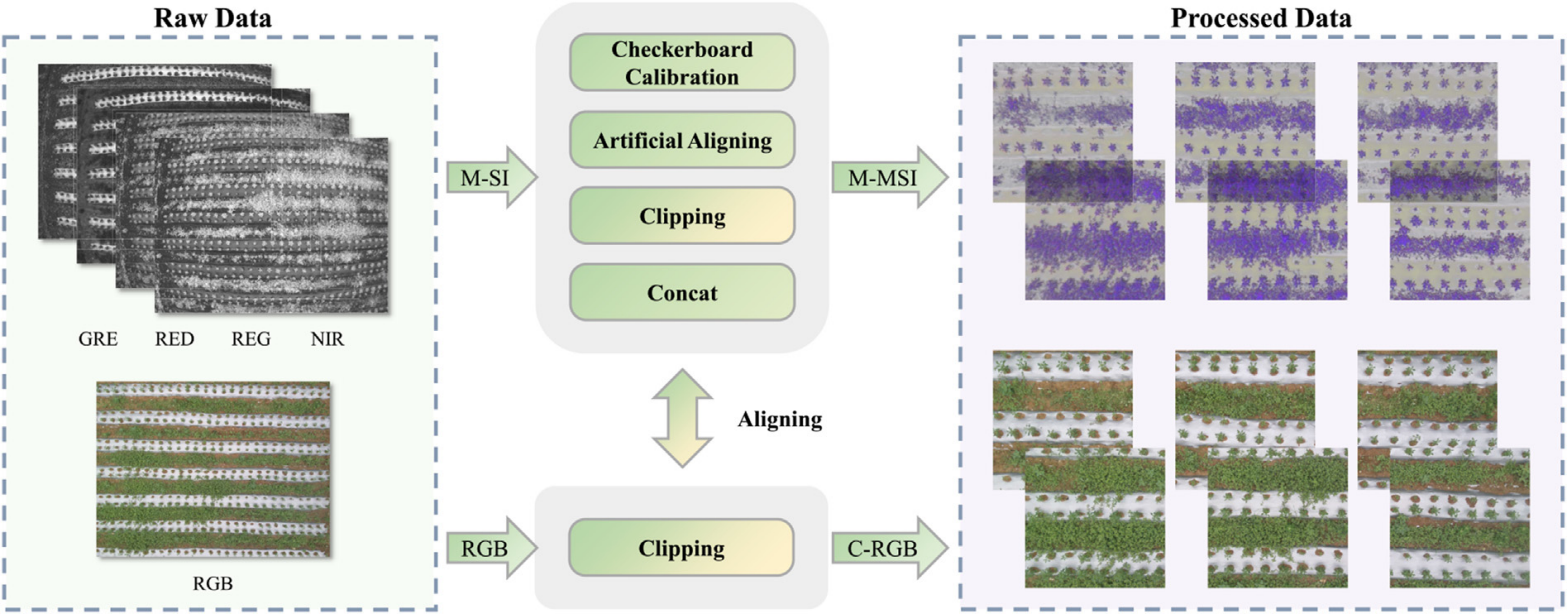
Data preprocessing flowchart. M ​− ​SI represents four single-channel spectral images in different wavelength bands, RGB represents the visible image, M-MSI represents the cropped 4-channel spectral image, and C-RGB represents the cropped visible image corresponding to the multispectral. This processing is performed to enrich the data for more robustness of our model.

As shown in Fig. 3, first, we adopt the checkerboard calibration method for distorted images to reduce the interference caused by fisheye distortion. Second, as the relative position of each camera lens is fixed, we use the manual alignment method to process a group of undistorted data to obtain the offset parameters between each lens and then batch process the remaining data. The manual alignment method employs an image overlay to judge the alignment of RGB images and multispectral images. We perform transparency adjustments on the RGB image and the multispectral image, respectively, and then superimpose them to observe the visual overlap and determine the accuracy of the alignment. Finally, six image blocks of size 340 ​× ​340 px are intercepted from the manually aligned data without overlapping. According to the common setting of the data set, we split the training and test sets in a ratio of 9:1. In addition, to quantify the segmentation performance of our method, the data in the test set are labeled with binarized segmentation results.

Due to the presence of fisheye aberrations, there are a few unavoidable errors in the processing of the checkerboard calibration and manual alignment, resulting in some irregular deviations in spatial dimensions between the RGB image and the processed multispectral image. The image errors have an impact on the SR process, and we refer to these spatial deviations as ”strong noise”. During model training, this error can have an impact on the training results, as well as positive and negative effects. With the increase of training epochs, the reconstructed spectral images will discard some information in order to achieve better metrics, resulting in incomplete information on the reconstructed spectral data. Therefore, the design of our method takes this into full consideration to improve the model's ability to cope with such errors and minimize the impact of these errors on this work.

### 2.3 Method

**Fig. 4:**
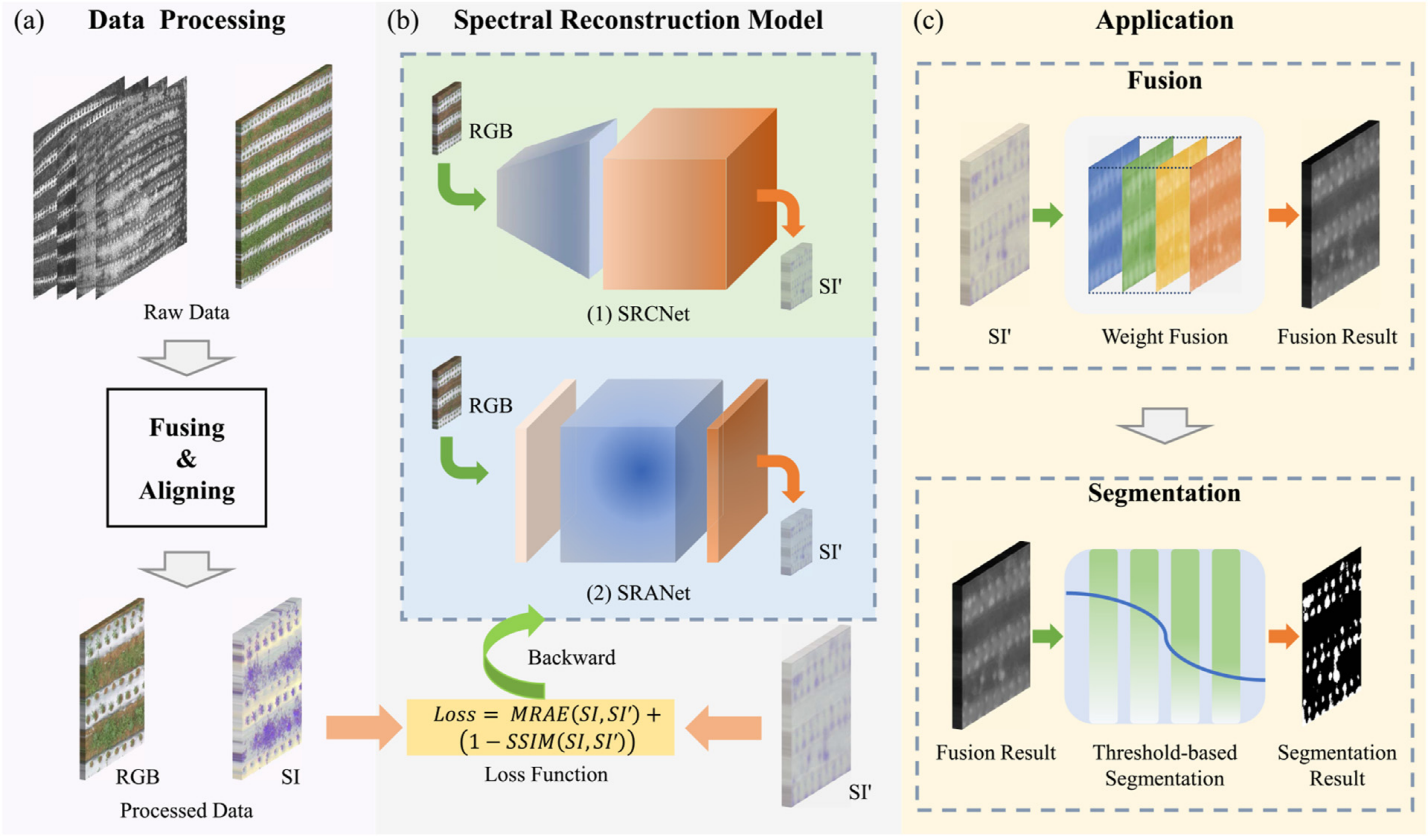
The main flowchart of our study, including the preprocessing of data, comparison between spectral reconstruction models, and application of the reconstructed data. *SI*′ represents the reconstructed spectral image. (a) Data processing including alignment, cropping, and other operations on the raw data to make it applicable to SR tasks. (b) Adaptation to data in multiple scenarios by using various types of SR models. In which only the training process requires the participation of spectral data. (c) Processing of SR results for application to relevant tasks.

Multispectral image data can capture the wealth of optical information available in the real world. In the field of agriculture and forestry, the vegetation index is typically calculated using spectral image data at different wavelengths to analyze various vegetation information [31]. In order to reduce the cost of spectral imaging, the spectral reconstruction task aims to reconstruct the corresponding spectral image using RGB images. In addition, to explore the application reliability of spectral reconstruction, we utilize the reconstruction results for application testing on downstream tasks. In this study, we propose two SR models for field image data, aiming to apply the rich spectral information conveniently to improve the accuracy of the analysis of field vegetation, as shown in Fig. 4. More specifically, we normalize the collected raw data through data processing. Then, the multispectral image mapped from RGB is obtained using our proposed spectral reconstruction model. Finally, spectral reconstruction data are fused using vegetation index theory and segmented with thresholds derived from information entropy.

#### 2.3.1 Spectral reconstruction

This study presents two different structure-based spectral reconstruction models, SRCNet and SRANet, to reconstruct the connection between spectral information and field phenotype. In addition, for comparison with our models, we select HRNet [32] (winner of the 2020 NTIRE spectral reconstruction competition [23]), the Snapshot Compressed Imaging (SCI) [33] based reconstruction model HDNet [34] and the natural image restoration model MPRNet [35], modify them to apply to our task.

**Fig. 5:**
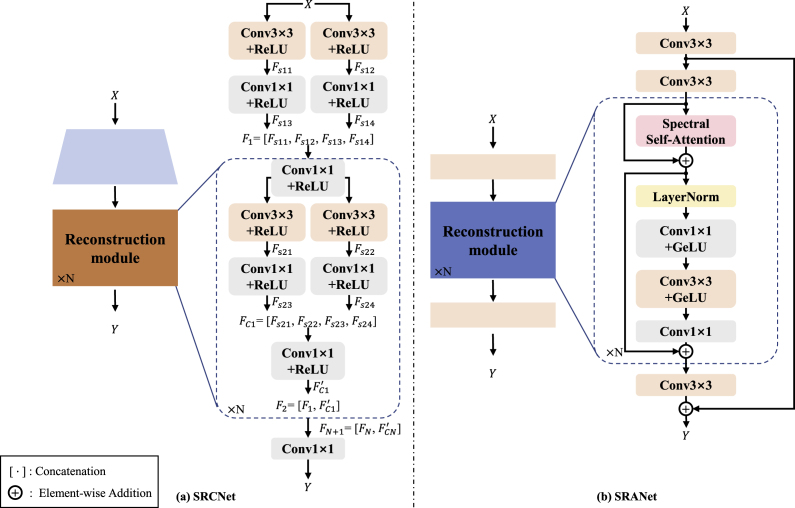
The structure of two SR models. Where *X* represents the input RGB image data and *Y* represents the reconstructed spectral image data.

As shown in Fig. 5(a), for SRCNet, the CNN-based spectral reconstruction model, we utilize convolutional blocks to compose the entire network. First, the model takes the input data for shallow extraction of features. Then, the features are processed by stacked convolutional blocks and activation blocks. Last, the convolutional block is utilized to reconstruct the target data. In order to ensure the completeness of the extracted features, we employ a large number of concatenation operations in the network to ensure that more intact features are retained during the model training process. In addition, the retention of more complete features through concatenation operations also serves to reduce the effect of spatial dimension misalignment caused by “strong noise”.

As shown in Fig. 5(b), for SRANet, the Attention-based spectral reconstruction model, we mainly use the attention module to compose the whole network. First, shallow feature extraction is performed by convolutional blocks. Then, a Transformer-like structure is utilized for feature processing, in which the spectral self-attention module is employed. After the stacking of multiple modules, the convolutional layer is finally employed to map to the target data. On the one hand, the introduction of the attention module enables the model to better focus on the relationship between spectral bands, which is beneficial to the reconstruction process. On the other hand, more attention to the relationship between spectral dimensions can ignore the spatial error caused by “strong noise” to a certain extent.

#### 2.3.2 Fusion strategy

Thanks to the spectral reconstruction model, we acquire spectral images reconstructed from RGB images. For extraction of salient features for each channel image, we perform feature fusion of the spectral reconstruction results. In general, processing multispectral image data in each waveband using VI is a common practice [36], and the normalized difference vegetation index (NDVI) [37] is one of the most widely used methods for analyzing plant growth conditions [38]. Therefore, based on the idea of NDVI, we get an index map to analyze the field situation by combining and calculating vegetation reflectance values at different wavelengths. Considering the quality of the spectral reconstructed images, our fusion strategy is not singularly similar to the VI to get more accurate segmented images of the field vegetation. To address this problem, we use two fusion strategies and test them. The aim is to select a fusion result that can effectively distinguish between field vegetation features and background features for the next stage of segmentation. Based on the above thoughts, we design the following two strategies to get the fused data *I*_*f*_ for application to the segmentation task.

Based on VI fusion, We apply the idea of VI to channel fusion according to the reconstruction results, which are defined as follows:

(1) $$I_{f_{v}}=\frac{C_{i}-C_{j}}{C_{i}+C_{j}},$$

where $I_{f_{v}}$ is the result of fusion based on VI strategies, *C*_*i*_ is the i-th channel data and *C*_*j*_ is the j-th channel data. We combine each channel of the reconstructed multispectral data based on the form of VI and select the combination with the best fusion effect.

Based on weight fusion, we weight and fuse the reconstructed multispectral data of each channel. More specifically, the band weights are decreased if the spectral reconstruction results are not good, and vice versa, the band weights are increased. The weight fusion method is as follows:

(2) $$I_{f_{w}}=\overset{n}{\underset{i=0}{\sum }}W_{i}*C_{i},$$

where $I_{f_{w}}$ is the result of fusion based on weights, *W*_*i*_ is the i-th channel weight and *C*_*i*_ is the i-th channel data, n represents the number of channels.

#### 2.3.3 Segmentation strategy

Traditional supervised segmentation methods require large amounts of labeled data to train the model, and data labeling is time-consuming and labor-intensive. To overcome the above problems, we consider a weakly supervised approach that does not require labeled segmentation data. Weakly supervised learning [39] is a machine learning method that utilizes limited, noisy, or indirectly supervised information to train a model when labeled data is incomplete, inaccurate, or costly. For the above two various fusion strategies, we use different methods to segment the fused data, respectively.

For the fusion strategy based on VI, we refer to the characteristics of the VI in the threshold setting. Normally, an NDVI index larger than zero means that there is vegetation cover, and we set the threshold around 0 to distinguish vegetation from the background. Since the band-based calculation of VI requires high-quality spectral images, we need to fine-tune the thresholds to achieve the target results. Moreover, we design threshold comparison experiments to select the result with the best effect in the experimental stage.

For the fusion strategy based on weight fusion, we explore a fixed-threshold segmentation strategy at the beginning of the experiment, and this setup only performs well on some of the data due to the one-sidedness of the fixed threshold. Therefore, we introduce the knowledge of information entropy to design an adaptive threshold [40]. Based on the information entropy of the fused images, the average value of the global features is made close to the threshold we set, so that our segmentation strategy can better adapt to the data of different scenes. Segmentation based on weight fusion strategy is defined as follows:

(3) $$\begin{array}{ll} & I_{f_{w}}>T_{s}\;\;\;as\;\;\;Vegetation,\\ & I_{f_{w}}<T_{s}\;\;\;as\;\;\;Background, \end{array}$$

where $I_{f_{w}}$ represents the reconstructed spectral data after fusion. *T*_*s*_ represents the threshold. *T*_*s*_ is defined as:

(4) $$T_{s}=\left\{\begin{array}{lll} & \varphi_{h}Avg\left(I_{f_{w}}\right)\;\;\;if & Avg\left(I_{f_{w}}\right)>S_{h},\\ & \varphi_{l}Avg\left(I_{f_{w}}\right)\;\;\;if & Avg\left(I_{f_{w}}\right)<S_{l}, \end{array}\right)$$

where *Avg*(⋅) represents the function to calculate the average value. *S*_*l*_ and *S*_*h*_ represent the lower and upper bound of the value, which is set manually. *φ*_*l*_ and *φ*_*h*_ represent constraint functions, which are used to constrain cases where the mean is too small or too large, respectively, and they are typically a number that indicates a weight.

### 2.4 Loss function

According to the loss functions commonly used in SR methods, we introduce three metrics to evaluate the quality of our reconstruction, respectively peak signal-to-noise ratio (PSNR), structural similarity (SSIM) [41], and mean relative absolute error (MRAE) [42]. In particular, SSIM and MRAE are utilized as the main metrics of our method, and these two metrics will be adopted as part of the loss function in the training process. The details of these formulas are as follows:

(5) $$MSE=\frac{1}{mn}\overset{m-1}{\underset{i=0}{\sum }}\overset{n-1}{\underset{j=0}{\sum }}{\left(X\left(i,j\right)-Y\left(i,j\right)\right)}^{2},$$

(6) $$PSNR=10\cdot \log_{10}\left(\frac{{\text{MAX}}^{2}}{\text{MSE}}\right),$$

where *m* and *n* are the number of rows and columns of the image, *X*(*i*, *j*) and *Y*(*i*, *j*) are the pixel values of the reconstructed and real data respectively, and *MAX* is the maximum possible value of the pixel value.

(7) $$SSIM=\frac{\left(2\mu_{X}\mu_{Y}+c_{1}\right)\left(2\sigma_{XY}+c_{2}\right)}{\left(\mu_{X}^{2}+\mu_{Y}^{2}+c_{1}\right)\left(\sigma_{X}^{2}+\sigma_{Y}^{2}+c_{2}\right)},$$

where *X* and *Y* represent the reconstructed image and the real image, respectively. *μ*_*X*_ and *μ*_*Y*_ are their means, *σ*_*X*_ and *σ*_*Y*_ are their standard deviations, *σ*_*XY*_ is their covariance. *c*_1_ and *c*_2_ are two constants to avoid the denominator being zero.

(8) $$MRAE=\frac{1}{n}\overset{n}{\underset{i=1}{\sum }}\frac{\left|X^{\left(i\right)}-Y^{\left(i\right)}\right|}{Y^{\left(i\right)}},$$

where $X^{\left(i\right)}$ represents the i-th pixel point of the reconstructed spectral data, and $Y^{\left(i\right)}$ represents the i-th pixel point of the real spectral data.

Combining the above metrics, we employ SSIM and MRAE as part of our loss function. The formula is as follows:

(9) $$Loss_{i}=M_{i}+\left(1-S_{i}\right),$$

where *M*_*i*_ is the MRAE of the i-th training epoch and *S*_*i*_ is the SSIM of the i-th training epoch. SSIM and MRAE are used as losses in order to measure the quality of the reconstructed image from a global image perspective, and PSNR is applied as one of the metrics to measure the quality of the reconstructed image from a visual sensory perspective.

### 2.5 Evaluation metrics

In the area of image processing, PSNR, SSIM, and MRAE are often used to evaluate the reconstruction quality of images [43]. We use the above three metrics to evaluate the spectral reconstruction quality to judge the effectiveness of our method.

Mean Intersection over Union (MIoU) [44] is widely used in traditional segmentation image quality evaluation metrics, and we also adopt this metric in our experiment. To compare with the real segmentation data image, we annotated the test dataset. We compare the field vegetation segmentation data obtained by our method with the real data and calculate its MIoU, MIoU is defined as follows:

(10) $$MIoU=\frac{1}{k}\overset{k}{\underset{i=1}{\sum }}\frac{P⋂G}{P⋃G},$$

where *P* and *G* stand for Prediction and Ground Truth (GT) respectively. There are *k* sets of data in the test dataset, statistics of the intersection and union regions between each set of prediction results and the true results, then the quality of segmentation results is measured by calculating MIoU.

## 3 Result

### 3.1 Qualitative analysis

**Fig. 6:**
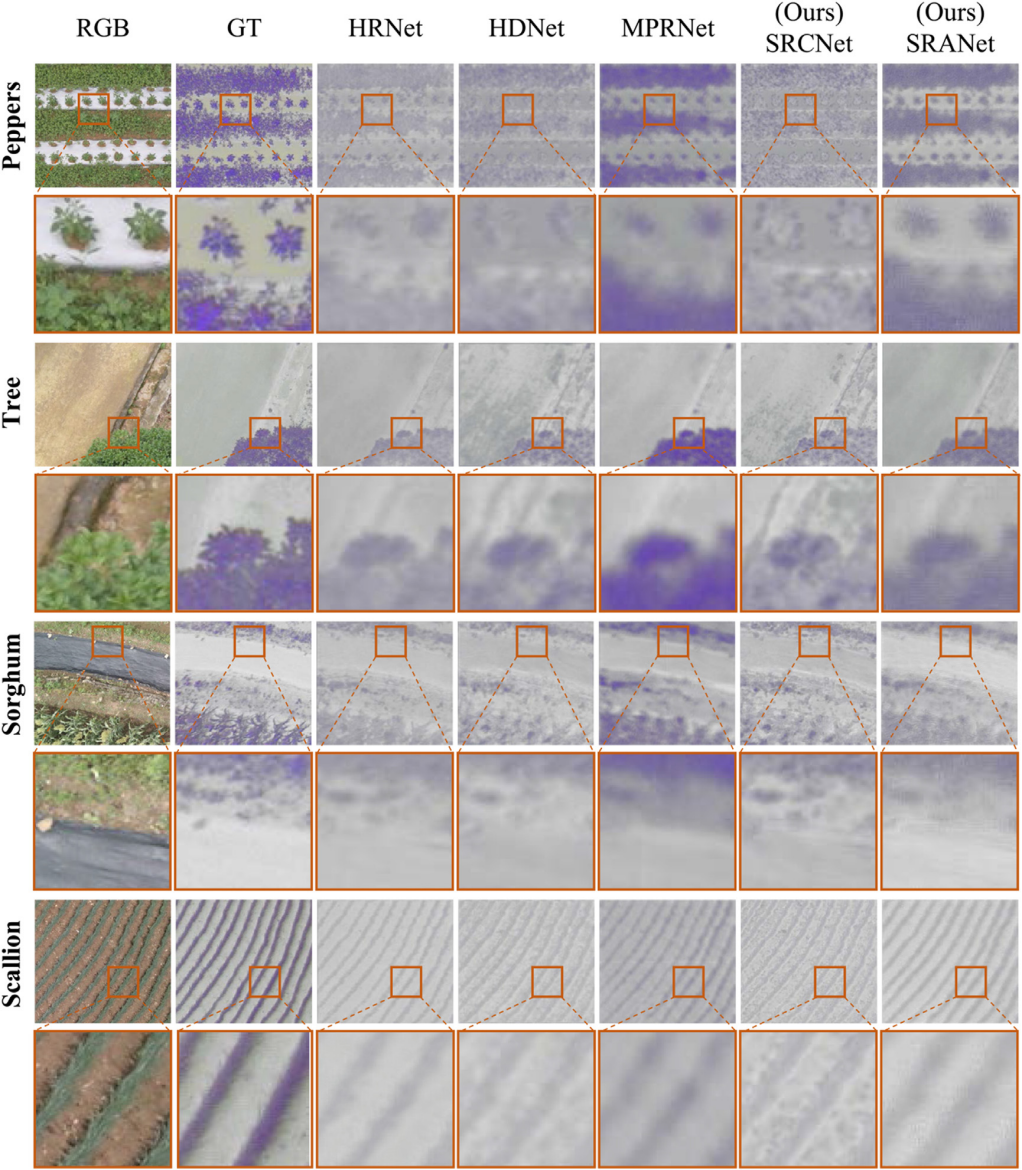
Visual comparison of the reconstructed spectral results. Colored boxes serve to highlight detailed areas of the reconstructed image to show the differences in these localized areas more clearly. GT represents the real spectral data.

To validate the effectiveness of our proposed methods, we compare the results of our models and other models on real field data. As shown in Fig. 6, we report the SR results of the five models under the ”strong noise” dataset, and the results are reflected in the following two aspects. On the one hand, compared with real spectral data, the reconstruction details of our methods are significantly superior to the results of other methods, especially in the structural information. On the other hand, SRCNet can obtain satisfactory results under different field scenes, which proves its generalization. To ensure the consistency of the experimental results we keep the same reconstruction parameter settings. The results demonstrate that the data generated by the SR model can show more potential for subsequent downstream tasks.

**Fig. 7:**
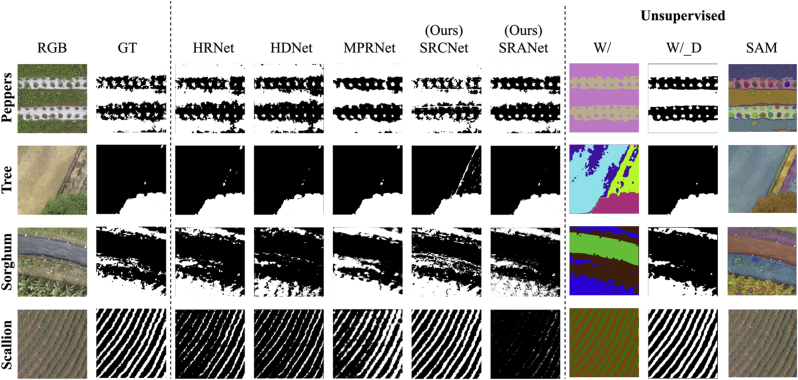
Comparison of the results of vegetation segmentation applications under the SR results of different models. The W/_D is the visualization of the vegetation and non-vegetation of the W/results, and the SAM results are directly using the ”Everything” option. GT represents the manually labeled real segmentation results.

**Fig. 8:**
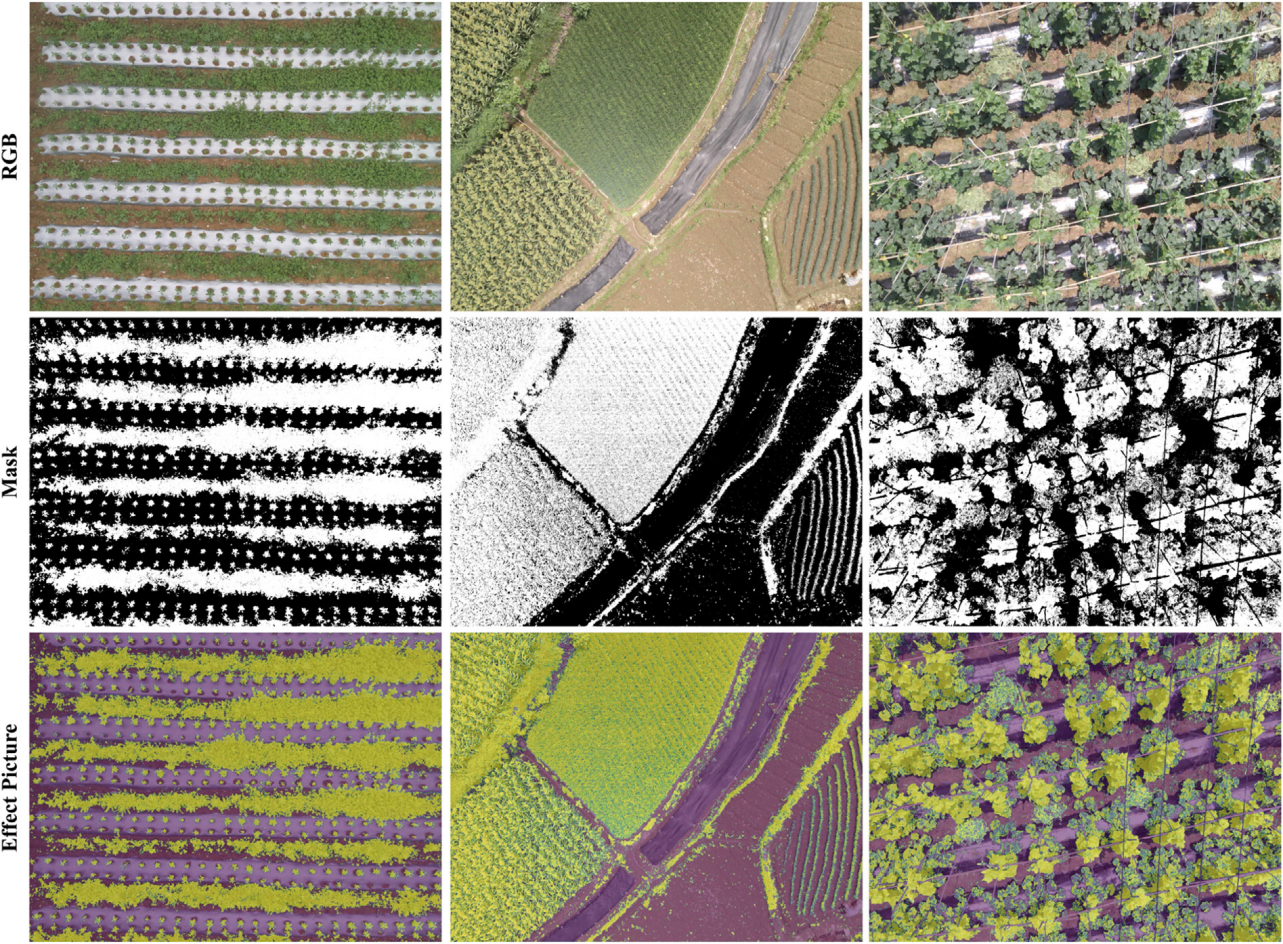
Complete field vegetation segmentation effect image. The first row represents the RGB image. The second row represents segmentation mask image. The third row represents the superimposed effect image of the RGB image and the segmentation mask.

As shown in Fig. 7, we fuse the reconstructed spectral data of each channel and further segment them, as well as report the segmentation results of the spectral data reconstructed by the five models. In addition to this, we also qualitatively compare with Segment Anything (SAM) [45], an existing unsupervised segmentation method. SAM is based on the Transformer architecture, which can automatically generate high-quality image segmentation masks based on simple prompts, and is widely used in the fields of image processing and image editing. To ensure the consistency of the experimental results we keep the same fusion strategy and segmentation parameter settings, uniformly adopt the fusion strategy based on the VI, and set the segmentation parameter to 0.2. The results reflect the following three points. First, the vertical comparison shows that our methods perform excellently on both dense vegetation and uniformly distributed vegetation. Second, the horizontal comparison shows that compared with existing unsupervised segmentation methods [46], our methods excel in the details of vegetation segmentation. Third, our methods are more accurate for vegetation segmentation in real field environments than the most prospective SAM [45] segmentation method. In addition, we validate the complete vegetation segmentation results experimentally. As shown in Fig. 8, we use SRCNet to segment full-size field images. The results show that our method can accurately segment real field vegetation in various complex scenes.

### 3.2 Quantitative analysis

To show the effectiveness of our methods, we quantitatively analyze the reconstruction results and their segmentation results from different aspects, respectively. For the SR results, we analyze the reconstruction metrics data by selecting the best results, as shown in Table 1. In addition, we perform the plant phenotype segmentation task based on the best reconstruction results, as shown in Table 2.

**Table 1:** Reconstruction metrics for the SR models.

Model	MRAE	SSIM	PSNR
SRCNet(Ours)	0.267	0.338	17.177
**SRANet(Ours)**	***0.230***	***0.385***	***18.134***
HRNet	0.260	0.380	17.407
HDNet	0.259	0.364	17.338
MPRNet	0.233	0.384	17.964

**Table 2:** Params-FLOPS-MIoU comparisons with SR models.

Model	Params(M)	FLOPS(G)	Pred	GT(%)		MIoU
				**Back**	**Veg.**	
**SRCNet(Ours)**	0.30	34.63	Back	89	5	***0.853***
			Veg.	11	95	
SRANet(Ours)	0.03	0.95	Back	85	8	0.799
			Veg	15	92	
HRNet	0.61	4.96	Back	85	6	0.809
			Veg.	15	94	
HDNet	0.01	1.08	Back	89	9	0.814
			Veg.	11	91	
MPRNet	0.06	41.23	Back	90	11	0.812
			Veg.	10	89	

The comparison of the experimental data shows that the spectral data reconstructed by our attention-based model outperforms the other models on MRAE, SSIM, and PSNR, as shown in Table 1. However, as shown in Table 2, a better SR metric does not mean that the results perform better in downstream tasks due to the effect of ”strong noise”.

For segmentation results in Table 2, we choose the best result achieved by each model in the selection of metrics, thus the results produced under different strategies. Table 2 reflects the following four points. First, segmentation based on spectral reconstruction is able to exhibit satisfactory results, which proves the feasibility of our methods. Second, our models achieve better segmentation results compared to other models. Third, our SRCNet is capable of achieving an MIoU of 0.853 in terms of segmentation results based on the use of VI as the fusion strategy, which indicates that the reconstruction results can be appropriately applied to other tasks. Finally, our methods are capable of achieving excellent results in various environments, demonstrating the high generalizability of our approach.

### 3.3 Reconstruction performance comparison

**Fig. 9:**
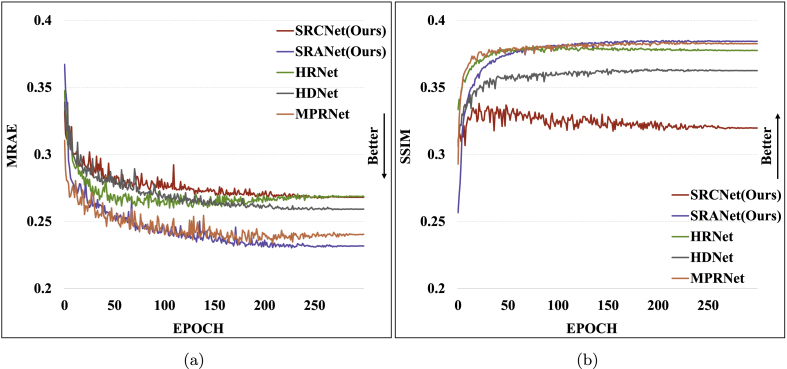
Comparison of model reconstructed spectral data metrics. (a) Trends in MRAE metrics of SR models. (b) Trends in SSIM metrics of SR models.

To analyze the sensitivity of different SR methods to real field data, we compare the performance of each model on our dataset. As shown in Fig. 9, we report the metrics of each SR method in the spectral reconstruction stage, where the attention-based model has more potential in the training process. The results show that the models have the same training process as the traditional deep learning models, and each metric trends better with the increase of the training epoch. This indicates that the reconstructed spectral data are closer to the real spectral data in the metrics, but the reconstructed data will discard some spatial information due to the presence of ”strong noise”.

### 3.4 Impact of fusion strategy on segmentation results

In order to obtain the best segmentation results, we adopt the VI-based fusion strategy and the weight-based fusion strategy, respectively. We set up the above two strategies separately to compare their segmentation performance. In particular, for the VI-based fusion strategy, we select bands 1 and 4 for the combined calculation. In terms of the weight-based fusion strategy, we set *W*_*i*_ to [−1.0, 0.0, 1.0, 1.0], while empirically setting the segmentation parameters *S*_*l*_ to 145, *S*_*h*_ to 150, *φ*_*l*_ to 1.15, and *φ*_*h*_ to 0.9. From Table 3, it can be concluded as follows. The weight-based fusion strategy is significantly lower than the VI-based fusion strategy due to the dataset contains mixed vegetation, using weight-based parameter values is only valid for some of the results and is not generalizable.

**Table 3:** Comparison of different fusion strategies based on different reconstruction methods.

Fusion Strategy	Model	Pred	GT(%)		MIoU	PA	MPA
			**Back**	**Veg**			
Based on VI	**SRCNet(Ours)**	Back	89	5	***0.853***	0.921	0.920
		Veg	11	95			
	SRANet(Ours)	Back	93	16	0.793	0.885	0.888
		Veg	7	84			
	HRNet	Back	93	15	0.802	0.890	0.893
		Veg	7	85			
	HDNet	Back	91	12	0.806	0.893	0.894
		Veg	9	88			
	MPRNet	Back	90	11	0.812	0.896	0.897
		Veg	10	89			
Based on Weight	**SRCNet(Ours)**	Back	88	32	***0.632***	0.775	0.781
		Veg	12	68			
	SRANet(Ours)	Back	90	36	0.615	0.763	0.772
		Veg	10	64			
	HRNet	Back	88	34	0.614	0.761	0.768
		Veg	12	66			
	HDNet	Back	87	31	0.631	0.774	0.780
		Veg	13	69			
	MPRNet	Back	89	33	0.627	0.772	0.779
		Veg	11	67			

### 3.5 Impact of segmentation parameters on segmentation results

**Fig. 10:**
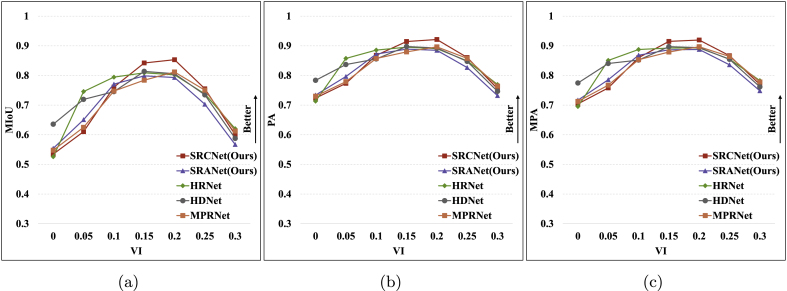
Comparison of VI-based segmentation on different parameter settings. (a) MIoU metrics. (b) PA metrics. (c) MPA metrics.

According to the different fusion strategies, we design different segmentation parameters to select the optimal results during the experiment. This experiment compares the field vegetation segmentation quality by adjusting the segmentation threshold parameters under the same SR method and the same fusion strategy. Since the VI-based strategy can achieve the best results, we choose it for this experiment. As shown in Fig. 10, segmentation results with VI parameter settings between 0.15 and 0.25 perform more superiorly in contrast to the conventional threshold of zero for NDVI, and the phenomenon we attribute to the poor quality of the reconstructed spectral results due to data effects.

### 3.6 Impact of epoch on segmentation results

**Fig. 11:**
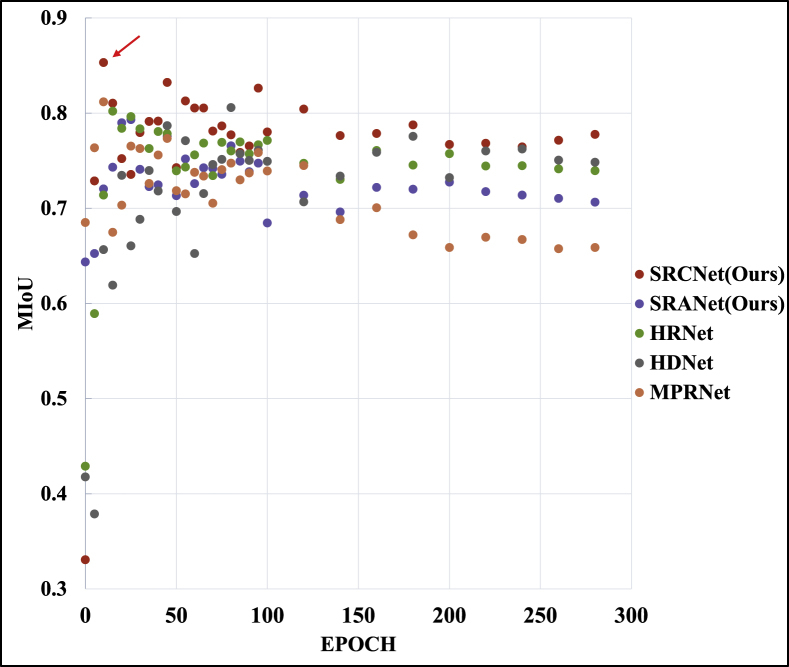
MIoU metrics of SR results for application to vegetation segmentation tasks under different epochs. The arrow points to the best segmentation result.

In section 3.3, we find that the reconstruction metrics gradually approach the ideal values as the training epochs progress. However, the segmentation results in the application of the segmentation task do not get better as the training metrics get better. We conclude that it is because the models are affected by ”strong noise” in the later stages of training, which leads to a spatial shift in the results. Therefore, we compare the model segmentation results of different training epochs to verify the negative impact of the ”strong noise” of the dataset on the subsequent downstream task applications, as shown in Fig. 11. First, the results show that the segmentation results are generally better in the early stage of training. Our trend is that in the early training epoch, the model learns the underlying inter-spectral connections without discarding too much information, i.e., it is less affected by ”strong noise”. Second, with the increase of training epochs, the results of each model slightly decrease and tend to stabilize. This is because as the reconstruction deepens, the influence of ”strong noise” gradually aggravates the spatial distortion of the reconstruction results, which in turn leads to a decrease in segmentation accuracy.

### 3.7 Ablation experiment

To validate the effectiveness of the proposed spectral reconstruction method in vegetation segmentation tasks, we design and conduct ablation experiments. In our methodological framework, RGB images are initially reconstructed into spectral images through SRCNet and SRANet, followed by the fusion of the reconstructed spectral images. The fused data is then segmented to achieve accurate field vegetation segmentation. In this experiment, we remove the spectral reconstruction module and apply our proposed fusion and segmentation strategy directly to the RGB images to assess the actual impact of spectral reconstruction on overall performance. Given that RGB images contain data from three channels (R, G, and B), we conduct experiments by pairing these channels in all possible two-channel combinations to compare results under our VI-based fusion strategy. As shown in Table 4, when the spectral reconstruction module is removed and RGB images are used directly for fusion and segmentation, the performance is noticeably inferior to that of the approach incorporating the spectral reconstruction module. The results indicate that the spectral reconstruction module plays a significant role in enhancing the accuracy of vegetation segmentation, effectively validating the critical importance of spectral reconstruction in our proposed method.

**Table 4:** Quantitative comparison of ablation experiments with spectral reconstruction module.

Model	Pred	GT(%)		MIoU	PA	MPA
		**Back**	**Veg.**			
**SRCNet(Ours)**	Back	89	5	***0.853***	0.921	0.920
	Veg.	11	95			
SRANet(Ours)	Back	93	16	0.793	0.885	0.888
	Veg.	7	84			
Based on VI(GB)	Back	68	78	0.264	0.432	0.448
	Veg.	32	22			
Based on VI(RB)	Back	68	69	0.311	0.483	0.496
	Veg.	32	31			
Based on VI(RG)	Back	76	60	0.579	0.567	0.579
	Veg.	24	40			
Based on Weight	Back	66	62	0.339	0.511	0.520
	Veg.	34	38			

In addition, in order to verify the positive effect of the multispectral image and the vegetation index on the experimental results, we also perform ablation experiments to remove these two components separately and compare their effects. As shown in Table 5, we compare the effects of not using multispectral images and VI-based strategies on the experimental results. As can be seen from the table, both spectral images and vegetation indices play an active role in the method, significantly improving the effectiveness of the segmentation task. Specifically, the segmentation accuracy of the model decreases significantly after removing the multispectral image, which indicates that the rich spectral information provided by the multispectral image is crucial for accurately recognizing and segmenting the vegetation. Similarly, the segmentation performance of the model decreases after not using the VI-based strategy. By quantifying the growth status and health of vegetation, the vegetation index can effectively improve the differentiation between vegetation and non-vegetation, and help the model recognize vegetation areas more accurately. Therefore, fully utilizing multispectral images and the theory of vegetation index can provide more valuable feature information for the vegetation segmentation task, which can better accomplish accurate vegetation segmentation.

**Table 5:** Quantitative comparison of ablation experiments with multispectral images and vegetation indices. MSI stands for multispectral image and VI stands for VI-based strategy.

MSI	VI	MIoU	PA	MPA
		0.339	0.511	0.520
*√*		0.632	0.775	0.781
	*√*	0.579	0.567	0.579
*√*	*√*	**0.853**	**0.921**	**0.920**

### 3.8 Open-source dataset experiment

**Fig. 12:**
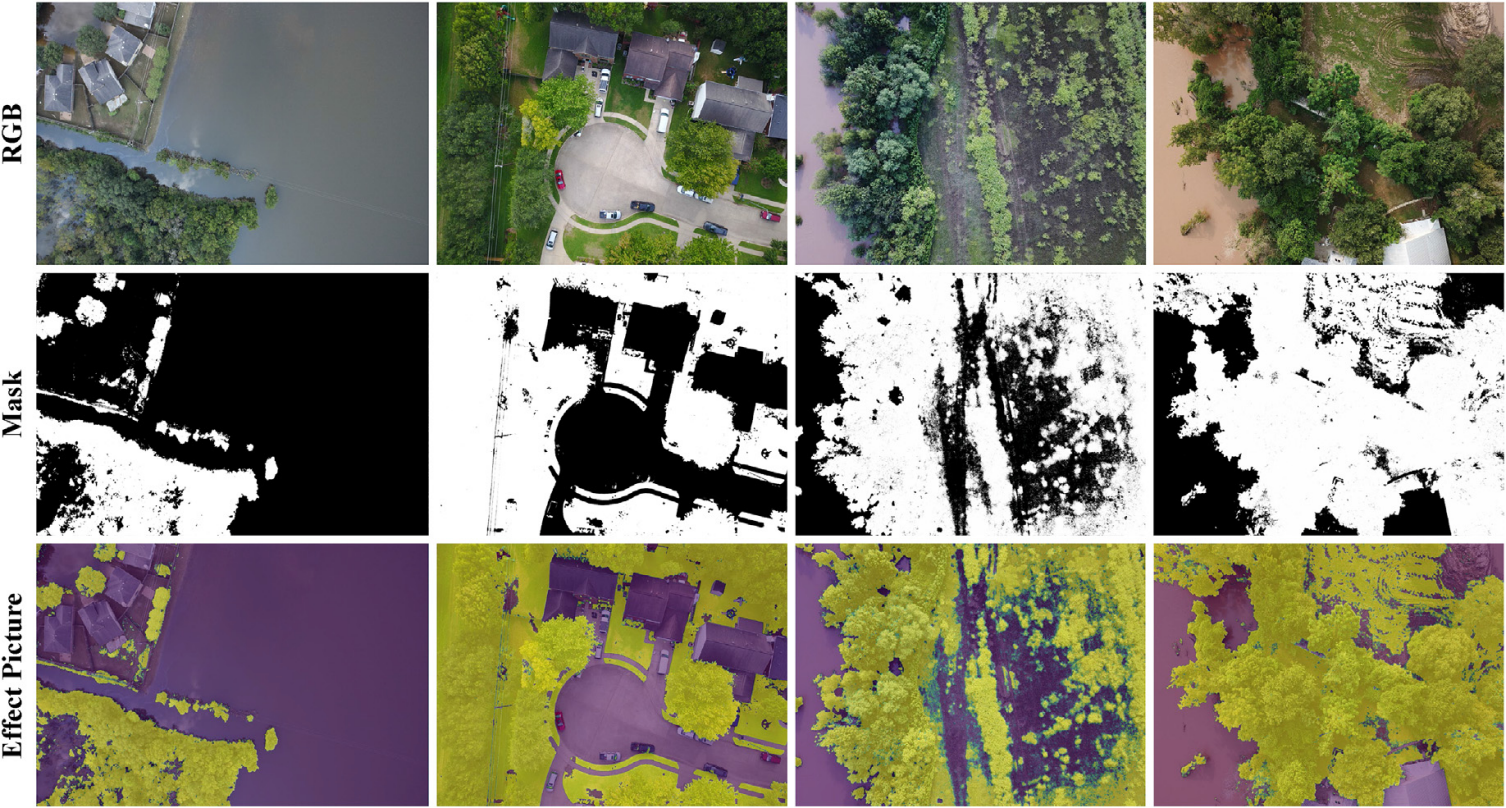
Qualitative results of segmentation experiments on FloodNet dataset. The first row represents the RGB image. The second row represents segmentation mask image. The third row represents the superimposed effect image of the RGB image and the segmentation mask.

To further validate the effectiveness of our method, we conduct comprehensive performance testing on an open-source dataset. Given the lack of real field drone image datasets, we select a similar drone dataset, FloodNet [47]. This dataset, collected using the DJI Mavic Pro quadcopter, encompasses various targets such as buildings, roads, floods, and vegetation, providing a diverse scene environment. When performing vegetation segmentation experiments on the FloodNet dataset, as shown in Fig. 12, SRCNet accurately identifies and segments vegetation areas, demonstrating significant segmentation effectiveness. This result not only highlights the superiority of our method on specific datasets but also validates its applicability in different environments. Our experimental findings further support the effectiveness of the proposed spectral reconstruction method, showcasing its potential value and wide applicability in real-world scenarios.

## 4 Discussion

### 4.1 Applications of spectral reconstruction

At present, there are many excellent methods for spectral reconstruction, but the application of the reconstruction results is rarely mentioned. Our study seeks to delve deeper into this issue and provide additional insights. More specifically, we apply the SR results to real-world downstream tasks and demonstrate the continued utility of reconstructed spectral data, even in cases where the reconstruction quality is not ideal. In this study, we rebuild spectral images from RGB images by spectral reconstruction technique, which results in improved data quality and provides richer information for subsequent studies. Spectral data are highly superior in vegetation phenotyping due to the richness of information they contain and their resistance to external interference [[48], [49], [50]]. Intuitively, if the SR method can generate spectral images that are extremely close to the truth, then the reconstructed images can be used directly for vegetation condition analysis. Nevertheless, due to the limitations of existing methods, even the current widely used deep learning methods still have slight errors in their results. Thus, we introduced the SR method into the vegetation segmentation task as a first step in processing the visible data, and then further processed the reconstructed data to obtain the desired data. Our study provides an idea for the practical application of SR results.

In the field of agriculture and forestry, the field vegetation segmentation map is extremely valuable data for use, which can effectively calculate the fractional vegetation cover (FVC) [51], leaf area index (LAI) [52,53], analyze the growth of field vegetation, assess the field environment. Furthermore, for some super-resolution segmentation maps, its features can be used to determine the development trend of field pests and diseases and timely manual intervention for control [54,55]. We are the first to propose the application of SR methods to vegetation segmentation tasks, and our method achieves satisfactory results with unlabeled datasets, which also demonstrates the potential of applying SR results to downstream tasks. In our method, the purpose of spectral reconstruction is to reduce the cost of spectral imaging. The image data acquired with commonly used or less expensive equipment is used to reconstruct or synthesize the corresponding spectral image, thus reducing the level of spectral imaging threshold. In addition, the application of reconstructed spectral images to downstream tasks enables them to take full advantage of the spectrum in a wider range of areas.

### 4.2 Discussion of experimental results

**Fig. 13:**
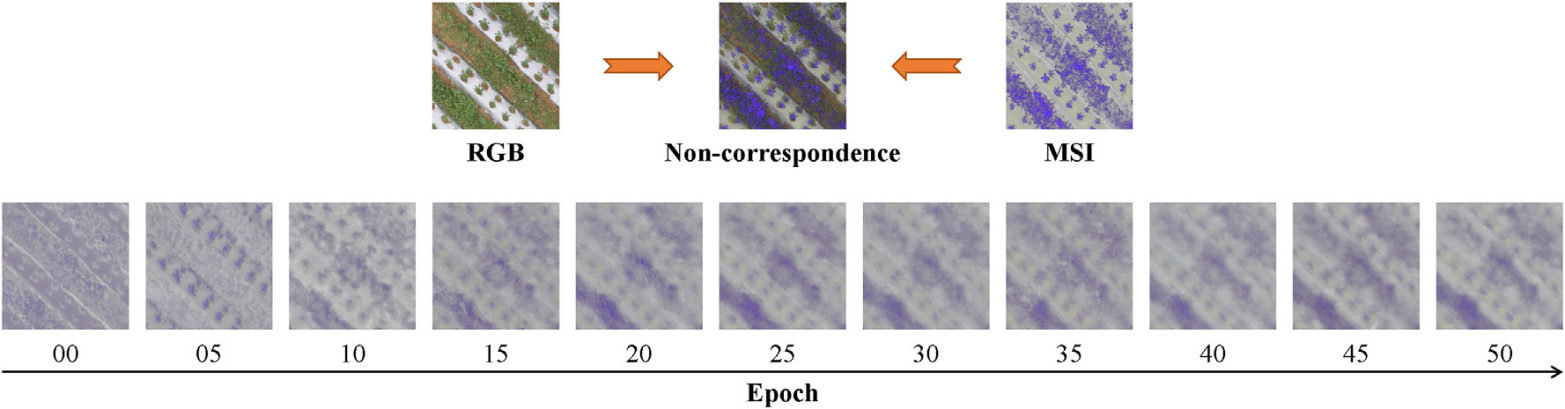
Visualization of “strong noise” in a dataset and its effect on spectral reconstruction.

The results of our experiments can truly show the phenotypic information of the vegetation in the field and can achieve satisfactory results. In the experiment, we choose the solution that generates the best results by comparing the experimental results under different methods. In the selection of certain options, we do not pick according to the conventional best choice. The aim of this is to minimize the effect of noise so that good results can be achieved in the subsequent tasks. For example, for the quality metrics of spectral reconstruction, a smaller MRAE and larger SSIM mean that the reconstructed data is closer to the target data. However, since the real field dataset we use has some unavoidable ”strong noise” in the process, the closer the reconstruction result is to the target data, the more it will be affected by the ”strong noise”. The impact of ”strong noise” on the spectral reconstruction results is shown in Fig. 13. As the training progresses, the reconstruction results are increasingly affected by strong noise, resulting in spatial offset. So we need to ”compromise” the result in the comparison process, that is, the generated data is as close as possible to the target data while being affected by the ”strong noise” as little as possible.

In the fusion and segmentation phase, we process the SR results to derive the target segmentation data. Due to the difference in sensitivity of each band spectrum to the same vegetation and background, we can segment the fused image by using threshold segmentation. Our proposed method can be effectively applied to a variety of UAV-based field data. In response to external influences such as different time phases, weather conditions, or some complex field environments, our method is still effective due to the introduction of spectral data. However, our approach exhibits certain limitations, including insensitivity to some minor target vegetation in the field or inability to balance the vegetation with substantial variations within the same scene, which is also a problem we need to pay attention to in future work.

### 4.3 Conclude and future work

In this paper, we propose a novel spectral reconstruction-based vegetation segmentation method, that can efficiently segment vegetation from different backgrounds without annotating the data. Among them, SRCNet performs well on the vegetation segmentation task, while SRANet is capable of reconstructing more realistic spectral images. But, there is still potential for improvement in our method. In future work, we will further optimize our approach and will use some of the data generated in this study to aid in the annotation. More specifically, we will label the corresponding areas pixel by pixel by category. In the model design, we will develop a segmentation model that can accurately distinguish various types of vegetation from each other in complex real-world environments.

## Author contributions

Z. Pei and Q. Wang conceived the idea and designed the experiments. Z. Pei and Xing. Wu conducted the experiments. Z. Pei and Xing. Wu contributed to the writing. Xing. Wu, Q. Wang, Xue. Wu and W. Guo contributed to the review. Q. Wang, Y. Xiao, P. Yu and Z. Gao provided funding support. All authors contributed equally to the writing of the manuscript.

## Data availability

Data is available at https://github.com/zhixunpei/VegSegment_SR.

## Funding

This research was supported by National Key R&D Program of China (2024YFD2001100, 2024YFE0214300), National Natural Science Foundation of China (No. 62162008), Guizhou Provincial Science and Technology Projects ( [2024]002, CXTD[2023]027), Guizhou Province Youth Science and Technology Talent Project ([2024]317), Guiyang Guian Science and Technology Talent Training Project ([2024] 2-15). The Talent Introduction Program of Guizhou University under Grant No. (2021)89.

## Declaration of competing interest

The authors declare that they have no known competing financial interests or personal relationships that could have appeared to influence the work reported in this paper.
